# The development of surgical risk score and evaluation of necrotizing soft tissue infection in 161 *Naja atra* envenomed patients

**DOI:** 10.1371/journal.pntd.0010066

**Published:** 2022-02-10

**Authors:** Chih-Sheng Lai, Po-Yu Liu, Chi-Hsin Lee, Cheng-Hsuan Ho, Wei-Ling Chen, Kuo-Lung Lai, Hung-Yuan Su, Wen-Loung Lin, Kuo-Chen Chung, Yi-Yuan Yang, Chung-Wei You, Kuang-Ting Chen, Yan-Chiao Mao

**Affiliations:** 1 Division of Plastic and Reconstructive Surgery, Department of Surgery, Taichung Veterans General Hospital, Taichung, Taiwan; 2 Division of Infection, Department of Internal Medicine, Taichung Veterans General Hospital, Taichung, Taiwan; 3 Rong Hsing Research Center for Translational Medicine, National Chung Hsing University; 4 School of Medical Laboratory Science and Biotechnology, College of Medical Science and Technology, Taipei Medical University, Taipei, Taiwan; 5 PhD Program in Medical Biotechnology, College of Medical Science and Technology, Taipei Medical University, Taipei, Taiwan; 6 Core Laboratory of Antibody Generation and Research, Taipei Medical University, Taipei, Taiwan; 7 Department of Emergency Medicine, Tri-Service General Hospital, Taipei, Taiwan; 8 School of Medicine, National Defense Medical Center, Taipei, Taiwan; 9 Psychiatry Department, Chiayi Branch, Taichung Veterans General Hospital, Chiayi, Taiwan; 10 Division of Clinical Toxicology, Department of Emergency Medicine, Taichung Veterans General Hospital, Taichung, Taiwan; 11 Division of Allergy, Immunology and Rheumatology, Department of Internal Medicine, Taichung Veterans General Hospital, Taichung, Taiwan; 12 Department of Emergency Medicine, E-Da Hospital and I-Shou University, Kaohsiung, Taiwan; 13 The School of Chinese Medicine for Post Baccalaureate, I-Shou University, Kaohsiung, Taiwan; 14 Taichung Wildlife Conservation Group, Taichung, Taiwan; 15 Division of Traumatology, Department of Emergency Medicine, Taichung Veterans General Hospital, Taichung, Taiwan; 16 Green Nature Experience, New Taipei, Taiwan; 17 Show Chwan Memorial Hospital, Changhua, Taiwan; Inkosi Albert Luthuli Central Hospital, SOUTH AFRICA

## Abstract

**Background:**

*Naja atra* bites cause wound necrosis, secondary infection, and necrotizing soft tissue infection (NSTI) requiring repetitive surgeries. Little information is known about the predictors for surgery after these bites.

**Materials and methods:**

We retrospectively evaluated 161 patients envenomed by *N*. *atra*, 80 of whom underwent surgery because of wound necrosis and infection. We compared the patients’ variables between surgical and non-surgical groups. To construct a surgical risk score, we converted the regression coefficients of the significant factors in the multivariate logistic regression into integers. We also examined the deep tissue cultures and pathological findings of the debrided tissue.

**Results:**

A lower limb as the bite site, a ≥3 swelling grade, bullae or blister formation, gastrointestinal (GI) effects, and fever were significantly associated with surgery in the multivariate logistic regression analysis. The surgical risk scores for these variables were 1, 1, 2, 1, and 2, respectively. At a ≥3-point cutoff value, the model has 71.8% sensitivity and 88.5% specificity for predicting surgery, with an area under the receiver operating characteristic curve of 0.88. The histopathological examinations of the debrided tissues supported the diagnosis of snakebite-induced NSTI. Twelve bacterial species were isolated during the initial surgery and eleven during subsequent surgeries.

**Discussion and conclusions:**

From the clinical perspective, swelling, bullae or blister formation, GI effects, and fever appeared quickly after the bite and before surgery. The predictive value of these factors for surgery was acceptable, with a ≥3-point risk score. The common laboratory parameters did not always predict the outcomes of *N*. *atra* bites without proper wound examination. Our study supported the diagnosis of NSTI and demonstrated the changes in bacteriology during the surgeries, which can have therapeutic implications for *N*. *atra* bites.

## Author summary

*N*. *atra* bites cause extensive wound necrosis and/or NSTI instead of neuroparalytic effect. We retrospectively evaluated 161 patients envenomed by *N*. *atra*, 80 of whom underwent surgeries because of wound necrosis and secondary infection. We found five variables—lower limb as the bite site, a ≥3 swelling grade, bullae or blister formation, GI effects, and fever that occurred rapidly after the bites—that were significant associated with surgery in the multivariate logistic regression analysis. The risk scores for these variables when integer the regression coefficients were 1, 1, 2, 1, and 2, respectively. The predictive value of these factors for surgery is acceptable, with a ≥3-point score. The common laboratory parameters did not always predict the outcomes of *N*. *atra* bites in the absence of proper wound evaluation. Deep tissue cultures and histopathological examinations supported the diagnosis of snakebite-induced NSTI. The bacterial species obtained during initial surgery differs from those obtained in subsequent surgeries, suggesting secondary colonization or nosocomial infections. These findings may be helpful for clinicians in better management and disposition of patients with *N*. *atra* bites.

## Introduction

Six medically important venomous snake species are found in Taiwan: *Trimeresurus stejnegeri stejnegeri*, *Protobothrops mucrosquamatus*, and *Deinagkistrodon acutus* in the Viperidae family and Crotalinae subfamily; *Daboia siamensis* in the Viperinae subfamily; and *Naja atra* and *Bungarus multicinctus multicinctus* in the Elapidae family [1]. *N*. *atra* also is widely found in southeastern China, North Laos, and North Vietnam [2]. In Taiwan, *N*. *atra* is the only cobra species, and its bite is uncommon (i.e., 6% [3]), except in the Central Plain region [1].

*N*. *atra* envenomation causes serious wound complications, including extensive wound necrosis; necrotizing soft tissue infection (NSTI); finger or toe wet gangrene necessitating distal extremity amputation; and systemic manifestations, such as gastrointestinal (GI) effects and fever [3,4]. Neuroparalytic effects were trivial in adults despite the presence of a short-chained alpha-neurotoxin in the venom [4,5]. Treatment consists of specific antivenom and antibiotics administration and surgical debridement when the necrosis margin is well demarcated [3,4,6].

Although the *N*. *atra* bite has been recognized as a risk factor for surgery among all venomous snakebites in Taiwan [7,8] and nearly half of the patients with bites underwent various surgeries because of wound necrosis and secondary infection [4], the risk factors for surgery remain less understood [9]. Therefore, to help first-line medical staff and clinicians better understand the risk factors for surgery and patients’ disposition, we retrospectively analyzed the data for 161 envenomed patients from the Taichung Veterans General Hospital (TCVGH) in central Taiwan.

## Materials and methods

### Ethics statement

This retrospective observational study was approved by the TCVGH Institutional Review Board (CE16225A and CE17219A). Informed consents were waived for such a retrospective study because of unidentifiable private information after obtaining approval from the Institutional Review Board.

### Study population

The patients were admitted to TCVGH between January 2001 and September 2016.

First we identified all possible cases of *N*. *atra* bites by searching the computerized database at TCVGH. Two authors independently reviewed the medical records of all patients with possible *N*. *atra* envenoming. Only definite and suspected cases were included for analysis [4]. We diagnosed a definite case by examining the culprit snake or laboratory testing of the venom [10–12] and a suspected case by having the patient identify the snake in a standard picture exhibited in the emergency department (ED). Some study participants overlapped with previous studies with a different reference period [3,4,13].

We extracted the following data: sex; age; body part bitten; first aid administered; comorbidities (e.g., diabetes mellitus, liver diseases [i.e., abnormal liver biochemistry, hepatic virus infection, or liver cirrhosis], vascular diseases (i.e., coronary artery disease [CAD] or cerebrovascular accident [CVA]), and malignancies); clinical manifestations; laboratory findings; and management details, including time elapsed between bite and antivenom administration, antivenom dosage, antibiotics administered, indications for surgery, time to initial and last surgery, surgery type, hospital stay length, and follow-up period [4,14]. We used consensus to resolve any disagreement between the authors during the data review and abstraction process.

### Definition of the variables

We extracted the variables from the patients’ medical charts. If no anomalies were mentioned in the case notes, we assumed that none were present.

The degree of swelling, which was modified from Blaylock’s classification [15], was categorized as Grade 1 (local swelling at the bite site), Grade 2 (swelling involving a whole hand or foot), Grade 3 (swelling from the hand to the forearm or from the foot to the leg), or Grade 4 (swelling extending to the whole arm, thigh, or the area above). Acute compartment syndrome (ACS) was diagnosed on the basis of typical signs and symptoms and operative notes of intracompartmental pressure >30 mmHg [16]. We classified the case with only clinical signs and symptoms suggestive of ACS but without documentation as suspected cases.

Local numbness was described as an effect that did not extend beyond the affected limb. Lymphangitis was clinically identified as a red line originating from the wound, whereas swollen tender lymph glands draining the affected area denoted lymphadenitis [4,14,17]. NSTI, including necrotizing changes in the layers of the soft tissue compartment and finger or toe wet gangrene were documented in the operations’ notes [4,18]. Fever was defined as a body temperature of ≥38°C measured using a tympanic thermometer. If a patient underwent surgery, only preoperative fever was included in analysis. GI effects included the presence of vomiting or diarrhea [4,11]. Ptosis was defined as partial and complete [19]; motor deficits were defined according to the modified Medical Research Council Scale for Muscle Strength [20].

All patients received blood tests on arrival at the hospital, and these data were repeatedly measured at the clinicians’ request thereafter. We analyzed the blood tests, including white blood cell (WBC) count, neutrophil-to-lymphocyte ratio (NLR), hemoglobin (Hgb), C-reactive protein (CRP), serum sodium, serum creatinine, blood glucose, and creatine kinase (CK), taken within 24 h post-bite and before surgery for those patients who underwent surgery [21,22]. Deep tissue culture was performed during surgical debridement, bullae fluid culture was performed with needle fluid aspiration, and blood culture was performed during febrile episodes. The culture sampling technique has been described previously [23]. Antibiotics administration was defined as any antibiotics used within 48 h after the bite.

### Statistical analysis

We compared the data between the definite and suspected cases as well as the surgical and non-surgical cases using the Mann–Whitney U test for the continuous variables and the chi-square test or Fisher’s exact test for the categorical variables. We developed a logistic regression model to predict surgery [21].

First, we included the factors including patients’ characters, clinical variables and laboratory findings, that had showed a significant association with surgery in the univariate logistic regression analysis in the multivariate backward elimination logistic regression analysis, with laboratory data obtained within 24 h post-bite and before surgery. We reported odds ratios (ORs) with corresponding 95% confidence intervals (CIs).

Second, to construct a diagnostic scoring system, we converted the predictors’ regression coefficients in the final model into integers. We expressed the score’s predictive accuracy as an area under the receiver operating characteristic (AUROC) curve. We used the Hosmer–Lemeshow analysis to test the model’s performance. We further examined the Laboratory Risk Indicator for Necrotizing Fasciitis (LRINEC) score [21] in those patients with NSTI [4].

We analyzed all data with SPSS version 26.0 (released 2019; IBM Corp., Armonk, NY, USA). A two-tailed *p*-value of <0.05 was considered statistically significant. If the Mann–Whitney U test was used, the statistical significance indicated a difference in groups and not association.

## Results

### Patient characteristics and clinical variables

A total of 170 patients with *N*. *atra* bites met the inclusion criteria. Nine were bites without noticeable signs or symptoms of envenoming; therefore, we analyzed data for 161 envenomed cases (Table 1). A total of 57 patients (35.4%) were definite cases, and 104 were suspected cases (Table 1, left columns); 80 patients (49.7%) had surgery, and 81 did not (Table 1, right columns). No significant variation was observed in the symptomatology between the definite and suspected cases.

**Table 1:** Characteristic data, clinical variables, and laboratory findings in 161 *Naja atra* envenomed patients.

	Definite case (n = 57)	Suspected case (n = 104)	*p*-value	Non-surgical case (n = 81)	Surgical case (n = 80)	Total cases (n = 161)	*p*-value^e^
Definite /Suspected cases, n (%)				26	31	57 (35.4)	
Male (%)	32	72	0.097	55	49	104 (64.6)	
Age (yr), mean ± SD	50.4 ± 17.7	53.2 ± 18.1	0.459	50.5 ± 17.4	53.9 ± 18.5	52.2 ± 18	
Body part bitten (%)			0.131				
Upper limb	25	60		53	32	85 (52.8)	0.001
Lower limb	30	43		26	47	73 (45.3)	0.001
Others (neck or trunk)	2	1		2	1	3 (1.9)	
First aid, n (%)							
Rope binding^a^	10	8	0.058	6	12	18 (11.2)	
Incision and suction	1	7	0.262	6	2	8 (5)	
Topical herbs	2	5	1	3	4	7 (4.3)	
Cold packs	6	5	0.199	4	7	11 (6.8)	
Alcohol ingestion	0	6	0.09	3	3	6 (3.7)	
Comorbidity (%)							
Diabetes mellitus	8	11	0.515	10	9	19 (11.8)	
Liver diseases	6	9	0.696	7	8	15 (9.3)	
Vascular diseases (i.e., CAD, CVA)	2	5	1	3	4	7 (4.3)	
Malignancies	2	1	0.286	1	2	3 (1.9)	
Clinical manifestations, n (%)							
Tissue swelling^b^			0.149				<0.001
Grade 1	7	14		21	0	21 (13)	
Grade 2	8	32		29	11	40 (24.8)	
Grade 3	24	35		21	38	59 (36.6)	
Grade 4	15	22		7	30	37 (23)	
Acute Compartment syndrome, suspected	3	5	1	1	7	8 (5)	0.034
Wound necrosis	41	69	0.466	32	78	110 (68.3)	<0.001
Bullae or blister formation	13	20	0.553	4	29	33 (20.5)	<0.001
Local numbness	13	25	0.907	19	19	38 (23.6)	
Lymphangitis or lymphadenitis	6	5	0.195	4	7	11 (6.8)	
Necrotizing soft tissue infection	28	44	0.406	0	72	72 (44.7)	<0.001
Necrotizing fasciitis	21	29	0.291	0	50	50 (31.1)	<0.001
Necrotizing adipositis	7	12	0.941	0	19	19 (11.8)	<0.001
Finger or toe wet gangrene	3	4	0.692	0	7	7 (4.3)	0.005
Fever (≥38°C)	22	33	0.359	7	48	55 (34.2)	<0.001
Gastrointestinal effects (i.e., vomiting or diarrhea)	22	30	0.206	11	41	52 (32.3)	<0.001
Ptosis or muscle weakness	5 (8.8)	3 (2.9)	0.133	1	7	8 (5)	0.034
Laboratory findings, median (IQR)							
Blood tests							
White blood cell counts, x 10^9^/L	8500 (7150–11970)	7900 (6420–10635)	0.285	7800 (6300–10500)	9200 (7100–11400)	8000 (6500–10785)	0.029
Neutrophil to lymphocyte ratio	4.7 (1.4–10.1)	3.8 (1.9–7.9)	0.966	2.6 (1.6–5.8)	7.3 (2.3–13.6)	3.9 (1.8–9.5)	0.001
Hemoglobin, g/dL	13.7 (12.3–15.2)	14 (12.6–15.1)	0.473	14 (12.5–15.1)	13.7 (12.5–15.1)	13.7 (12.5–15.1)	
C-reactive protein, mg/dL	0.15 (0.05–0.8)	0.46 (0.1–1.13)	0.109	0.2 (0.06–0.8)	0.48 (0.09–3.32)	0.28 (0.09–1)	
Serum sodium, mEq/L	140 (139–143)	141 (138–143)	0.786	140 (139–142)	141 (138–143)	140 (139–143)	
Serum creatinine, mg/dL	0.8 (0.7–1.0)	1 (0.8–1.1)	0.006	1 (0.8–1.1)	0.9 (0.7–1.1)	1 (0.8–1.1)	
Blood glucose, mg/dL	120 (103–143)	118 (104–141)	0.929	118 (103–142)	123 (106–139)	119 (103–141)	
Blood creatine kinase, U/L	147 (86–312)	162 (112–277)	0.523	134 (95–217)	180 (115–473)	159 (102–284)	0.018
Bacterial culture, case number							
Deep tissue culture	11	22	0.838				
Positive aerobic culture	10 / 11^c^	20 / 22	1				
Positive anaerobic culture	1 / 6	0 / 13	0.316				
Polymicrobial (≥2 pathogens)	5	8	0.714				
Positive blood culture	1 / 19	2 / 24	1				
Positive bullae fluid culture	1 / 1	ND^d^					

a: included any form of rope, rubber band, or towel/clothes bindings

b: 3 bitten site other than limbs and 1 swelling grade missed were excluded

c: 10 of 11 patients had positive cultures

d: not done

e: only values of statistical significance were listed.

Local swelling and pain were observed in all envenomed patients. Suspected ACS was found in eight patients (5%); seven of these received surgery because of concomitant wound necrosis and suspected secondary infection, and one patient did not have wound necrosis and refused surgery, ultimately recovering well after antivenom therapy. Wound necrosis; bullae or blister formation; local numbness; lymphadenitis or lymphangitis; fever; GI effects including vomiting or diarrhea; and neurological effects such as partial ptosis or equivocal, barely detectable weakness (Grade 5–) occurred in 68.3%, 20.5%, 23.6%, 6.8%, 34.2%, 32.3%, and 5% of the patients, respectively. NSTI was identified in 44.7%.

The median time elapsed between bite and GI effects and that between bite and fever was 2.3 h [interquartile range (IQR), 1–4.2 h) and 22 h (IQR, 13–37.6 h), respectively. Bullae or blisters in patients appeared before surgery, whereas swelling in patients reached its highest grade but not necessarily most swelling before surgery if patients underwent surgery. No deaths happened during the study period.

### Patient laboratory findings

The blood WBC, NLR, Hgb, CRP, serum sodium, creatinine, blood glucose, and CK levels could be obtained in 142, 138, 142, 83, 139, 142, 103, and 129 patients, respectively. No snakebite-related systemic bleeding or coagulopathy occurred.

Aerobic or anaerobic deep tissue cultures were performed in 33 of the 72 patients with NSTI, including a positive result in 30 of the 33 patients who received an aerobic culture and in 1 of the 19 patients who received an anaerobic culture (Table 1). Thirteen patients had polymicrobial infections. Blood culture was performed in 43 patients, and 3 had a positive result, including *Shewanella algae*, *Bacteroides fragilis*, and an unidentified gram-positive bacillus. The antibiotics administered in the three patients before obtaining the positive blood culture were cefazolin, gentamicin, and piperacillin/tazobactam in Patient 1; cefazolin, gentamicin, and ampicillin/sulbactam in Patient 2; and ampicillin/sulbactam in Patient 3. Bullae fluid culture was available in one patient in whom a positive blood culture (*S*. *algae*) was obtained at the same time; the isolated pathogens were *S*. *algae* and *Morganella morganii*.

Eighteen bacterial species were isolated in the deep tissue cultures (Table 2). These cultures were further grouped based on their acquisition during the initial or subsequent surgeries. During the initial surgery, 22 patients and 1 patient had a positive report in 24 aerobic and 16 anaerobic culture samplings, respectively. The most common isolated pathogens during the initial surgery were *Enterococcus* sp. and *M*. *morganii*, which also contributed to the polymicrobial infection. In addition, Group A *Streptococcus* sp., *S*. *putrefaciens*, *Aeromonas hydrophila*, *Citrobacter freundii*, *Proteus mirabilis*, *Yokenella regensburgei*, and *B*. *fragilis* were seen only in the initial surgery. Coagulase-negative *Staphylococcus* sp., *Staphylococcus aureus*, *Enterobacter cloacae*, *Providencia rettgeri*, *Pseudomonas aeruginosa*, and *Stenotrophomonas maltophilia* were seen only in the subsequent surgeries. The antibiotics administered before the initial surgery to obtain the culture are summarized in Table 2.

**Table 2:** Deep tissue cultures obtained during initial and subsequent surgeries.

	Culture obtained during initial surgery		Culture obtained during subsequent surgeries	
	Case numbers	Polymicrobial, case numbers	Case numbers	Polymicrobial, case numbers
Aerobic culture (positive case / total case)	22 / 24^a^		10 / 14	
Anaerobic culture (positive case / total case)	1 / 16		0 / 6	
Polymicrobial infection (≥2 pathogens)	9		5	
Gram-positive bacteria				
*Enterococcus* sp.	12	6	4	2
Group A, *Streptococcus* sp.	1	1		
Coagulase-negative *Staphylococcus* sp.			2	1
*Staphylococcus aureus*			2	
Gram-negative bacteria				
*Morganella morganii*	11	5	2	1
*Serratia marcescens*	2	2	1	1
*Shewanella putrefaciens*	2	2		
*Aeromonas hydrophila*	1	1		
*Citrobacter freundii*	1	1		
*Enterobacter cloacae*			1	1
*Proteus vulgaris*	1	1	3	2
*Proteus penneri*	1	1	1	
*Proteus mirabilis*	1	1		
*Yokenella regensburgei*	1			
*Providencia rettgeri*			1	1
*Pseudomonas aeruginosa*			2	1
*Stenotrophomonas maltophilia*			1	1
Anaerobic bacteria				
*Bacteroides fragilis*	1	1		
Antibiotics administered before initial surgery, in descending order			NA^b^	
Ampicillin/sulbactam	10			
Gentamicin	9			
Cefazolin	8			
Amoxicillin/clavunate	6			
Moxifloxacin	5			
Metronidazole	3			
Oxacillin	2			
Penicillin	2			
Ceftriaxone	2			
Cefotaxime	2			
Ampicillin	1			
Piperacillin/tazobactam	1			
Cefoperazone/sulbactam	1			
Levofloxacin	1			

a: 22 of the 24 patients had positive cultures

b: NA: not analyzed.

### Patient management and outcomes

A total of 137 patients (85.1%) received the specific antivenom within 6 h after the bite (Table 3). The exact time of first antivenom administration was available in 88 cases; and the median time elapsing between the bite and administration of initial antivenom dose was 2.6 h (IQR, 1.4–5.6 h), and the median total dose was 10 vials (IQR, 6–14 vials). A total of 150 patients (93.2%) received antibiotics.

**Table 3:** Management of 161 *Naja atra* envenomed patients.

	Definite case (n = 57)	Suspected case (n = 104)	*p*-value	Non-surgical case (n = 81)	Surgical case (n = 80)	Total cases (n = 161)	*p*-value^e^
Specific antivenom administration							
Time elapsed between bite to initial antivenom dose, n (%)			0.497				
<6 h	47	90		68	69	137 (85.1)	
6–12 h	5	5		6	4	10 (6.2)	
>12 h	5	8		6	7	13(8.1)	
Median in h (IQR)	2.7 (1.4–6.4)	2.5 (1.4–5.5)	0.65	2.6 (1.4–4.5)	2.7 (1.4–7.2)	2.6 (1.4–5.6)	
Total antivenom dose in vials, median (IQR)	10 (7–16)	10 (6–13)	0.286	6.5 (4–12)	12 (8.25–16)	10 (6–14)	<0.001
Antibiotics administration within 48 h post-bite, n (%)	55	95	0.331	71	79	150 (93.2)	0.005
Operation case, n (%)	31	49	0.378				ND^f^
Time elapsed between bite to initial surgery in days; median (IQR)	2.9 (1.6–5)	3.1 (1.9–6.2)	0.352		3 (1.9–5.4)	3 (1.9–5.4)	
Time elapsed between bite to last surgery in days; median (IQR)	20.2 (17.4–41.6)	26 (19–42.4)	0.394		25.7 (18–41.6)	25.7 (18–41.6)	
Debridement, n (%)	23	40	0.582	0	63	63 (39.1)	
Fasciotomy/Fasciectomy	16	16	0.037	0	32	32 (19.9)	
Finger or toe amputation	3	4	0.692	0	7	7 (4.3)	
STSG/FTSG^a^	18	24	0.215	0	42	42 (26.1)	
Flap^b^	2	8	0.495	0	10	10 (6.2)	
Others^c^	1	8	0.167	0	9	9 (5.6)	
Number of surgeries per patient, n (IQR)	3 (2–4.3)	3 (2–4)	0.506			3 (2–4)	
Hospital stay in days, median (IQR)	9 (2.7–25.6)	5.4 (1.8–19)	0.124	2.1 (0.9–4.9)	23.4 (9.7–30.8)	6.8 (2–23.8)	<0.001
Outpatient follow-up case, n (%)	41	65	0.228	44	62	106 (65.8)	0.002
Follow-up in days, median (IQR)^d^	40 (14–71)	37 (15–68.5)	0.704	14 (7.25–31.8)	55.5 (35.8–90.3)	37 (14–70)	<0.001

a: split-thickness skin grafting/full-thickness skin grafting

b: free or rotational flap

c: staged excision, division of flap, revision of flap, or revision of scar

d: from day of the bite to the last day of follow-up

e: only values of statistical significance were listed

f: not done.

The surgical indications were wound necrosis and secondary infection in all 80 patients. The initial surgery was performed at a median of 3 days (IQR, 1.9–5.4 days), whereas the last surgery was performed at a median of 25.7 days (IQR, 18.0–41.6 days) after the bite. The median hospital stay was 6.8 days (IQR, 2.0–23.8 days) for all patients; the stay was 2.1 days (IQR, 0.9–4.9 days) for the non-surgical patients and 23.4 days (IQR, 9.7–30.8 days) for the surgical patients. The median follow-up period was 37 days (IQR, 14–70 days) (Table 2). Surgical pathology was obtained in 14 patients, and acute necrotizing inflammation was commonly mentioned (Table 4).

**Table 4:** Clinical manifestations and histopathological findings of the debrided tissues in 14 envenomed patients.

Case number	Sex	Age	Body part bitten	Clinical manifestations	Type of surgery	Time elapsed between bite and surgery in days	Pathological findings at the time of surgery	Hospital stay in days
1	Female	69	Finger	Swelling, pain, suspected acute compartment syndrome, wound necrosis, vomiting, fever; necrotizing fasciitis (NF)	Fasciotomy	0.4		44.7
					Debridement	3.1		
					Debridement	6		
					Debridement and finger amputation	10.9	Finger: necrotizing inflammation of soft tissue and joint	
					Debridement	17		
					Debridement	23.9		
					STSG^a^	33.7		
2	Male	44	Calf	Swelling, pain, wound necrosis, fever; necrotizing adipositis	Debridement	4.1	Soft tissue: necrotizing suppurative inflammation of skin and soft tissue	4.8
3	Male	70	Finger	Swelling, pain, wound necrosis, local numbness	Finger amputation	1.2	Finger: necrosis	5.8
4	Male	38	Hand	Swelling, pain, suspected acute compartment syndrome, wound necrosis, local numbness, bullae and blister formation, fever, NF	Fasciectomy	1.2		36.5
					Fasciotomy	13.1	Blood vessel: acute vasculitis and thrombosis	
					Free flap transplantation	22.2		
					Revision of scar and debulking surgery	105.4		
					Revision of scar	229.1		
5	Female	72	Toe	Swelling, pain, wound necrosis, vomiting, diarrhea, fever, NF, toe gangrene	Debridement	5	Soft tissue: acute necrotizing inflammation, acute and chronic inflammation involved skin, dermis, subcutis. Perivascular lymphocytic infiltration	29.8
					Toe amputation, rotational flap, STSG	18.9	Toe: skin ulceration, chronic inflammatory cells infiltration and fibrosis	
6	Male	82	Finger	Swelling, pain, wound necrosis, bullae and blister formation, diarrhea	Debridement	4.7		20.8
					Finger amputation	11.7	Finger: acute necrotizing inflammation	
7	Male	58	Finger	Swelling, pain, wound necrosis, bullae and blister formation, diarrhea, fever; necrotizing adipositis, finger gangrene	Debridement and finger amputation	4.8	Finger: gangrene	24
					Debridement	14.8		
					Debridement and STSG	18.7		
8	Female	50	Toe	Swelling, pain, wound necrosis, bullae and blister formation, vomiting, fever, NF	Fasciotomy	2	Soft tissue: acute necrotizing inflammation with abscess formation	31.5
					Debridement	5.6		
					Debridement	14.5		
					STSG	19.9		
9	Male	35	Forearm	Swelling, pain, wound necrosis, bullae and blister formation, vomiting, diarrhea, fever, NF	Fasciotomy	5	Soft tissue: acute necrotizing and suppurative inflammation	25.2
					Debridement	11.1		
					STSG	17.8		
10	Female	62	Toe	Swelling, pain, wound necrosis, lymphangitis and lymphadenitis, vomiting, fever, NF	Fasciotomy	8.2	Soft tissue: acute necrotizing inflammation	32.9
					Debridement	12.1		
					Debridement	21.2		
					STSG	25.8		
11	Female	38	Toe	Swelling, pain, wound necrosis, vomiting, NF	Fasciotomy	8.7	Soft tissue: acute and chronic inflammation, recent hemorrhage, congestion and focal fat necrosis of subcutaneous tissue, supraepidermal cleft with hemorrhage and perivascular lymphocytic infiltration in the dermis	26.1
					Debridement	14.5		
					STSG	25.5		
12	Male	85	Finger	Swelling, pain, wound necrosis, bullae and blister formation, vomiting, diarrhea; NF, finger gangrene	Fasciectomy	3.2	Soft tissue: acute necrotizing and suppurative inflammation with abscess formation	23.9
					Debridement	9.9		
					Finger amputation	18	Finger: ulcer, acute necrotizing and chronic xanthomatous inflammation with recapillarization and vasculitis	
13	Male	45	Finger	Swelling, pain, wound necrosis, local numbness, bullae and blister formation, fever, finger gangrene	Finger amputation at distal interphalangeal joint (2nd finger)	8.8	Finger (distal): acute necrotizing inflammation and gangrene	23.6
					Debridement and finger amputation at proximal interphalangeal joint	10.8		
					Debridement	15.8		
					Removal of finger stump	21.6	Finger (proximal): acute inflammation of deep soft tissue	
14	Female	84	Calf	Swelling, pain, wound necrosis, lymphangitis, diarrhea, fever; NF	Fasciotomy	2.1	Soft tissue: acute inflammation	54.8
					Debridement	6.2		
					Debridement	9.2		
					Debridement	14.1		
					Debridement	16.9		
					Rotational flap and STSG	22.3		
					Debridement	29.1		
					STSG	49.1		

a: split-thickness skin graft.

### Statistical findings

From the univariate logistic regression analysis, the significant clinical variables for surgery were lower limb as the bite site, swelling grade, bullae or blister formation, GI effects, and fever, and the significant laboratory variables were WBC and NLR (Tables 5 and S1). Because no surgery was performed in patients with Grade 1 swelling, we grouped the swelling grade into a dichotomous variable for better comparison (i.e., Grade ≥3 and <3). Wound necrosis is the indication for surgery but not predictor; therefore, it was not entered into the logistic regression model. As a result, we found that lower limb as the bite site, a ≥3 swelling grade, bullae or blister formation, GI effects, and fever were significant in the multivariate backward elimination logistic regression analysis. The WBC and NLR were potential confounders. We then fit these significant variables into a final multivariate logistic regression model containing the entire study population. The regression coefficients of the predictors in the final model were 1.4, 1.4, 2.1, 1.1, and 1.7, respectively. The scores were 1, 1, 2, 1, and 2, respectively, when converting the regression coefficients. No interaction was observed between any two of the five variables.

**Table 5:** Risk factors for surgery in logistic regression and the association between clinical variables.

	Surgery OR (95% CI), *p*-value					Clinical variables crude OR (95% CI), *p*-value				
	Crude OR	Adjust OR (backward elimination method)	Regression coefficient	Adjust OR (enter method)	Regression coefficient	Lower limb as bite site	Swelling grade ≥3	Bullae or blister formation	GI effects	Fever
Lower limb as bite site	3 (1.6–5.7) 0.001	3.3 (1.2–9.2) 0.025	1.2	4 (1.6–9.9) 0.003	1.4	ND^b^	1.7 (0.9–3.3) 0.103	1.2 (0.6–2.6) 0.652	1.1 (0.5–2) 0.886	2.5 (1.3–4.9) 0.007
Swelling grade (1–4)	4.4 (2.7–7.2) <0.001	--	--	--	--	1.1 (0.8–1.6) 0.445	ND	2.8 (1.7–4.8) <0.001	5.4 (3–9.7) <0.001	4.8 (2.8–8.3) <0.001
<3	Reference	Reference	--	Reference	--	Reference	--	Reference	Reference	Reference
≥3	11 (5–24.3) <0.001	4.5 (1.4–14.2) 0.011	1.5	3.9 (1.5–10.4) 0.006	1.4	1.7 (0.9–3.3) 0.103	ND	4.4 (1.6–12.1) 0.004	13.7 (4.6–40.7) <0.001	22.4 (6.6–76.6) <0.001
Bullae or blister formation	10.8 (3.6–32.6) <0.001	11.4 (2.7–47.9) 0.001	2.4	8.1 (2.2–30.2) 0.002	2.1	1.2 (0.6–2.6) 0.652	4.4 (1.6–12.1) 0.004	ND	2.8 (1.3–6.1) 0.01	4.7 (2.1–10.7) <0.001
GI effects	6.7 (3.1–14.5) <0.001	4.2 (1.4–12.5) 0.011	1.4	2.9 (1.0–8.3) 0.042	1.1	1.1 (0.5–2) 0.886	13.7 (4.6–40.7) <0.001	2.8 (1.3–6.1) 0.01	ND	6.7 (3.2–14) <0.001
Fever	16.1 (6.6–39.6) <0.001	6.8 (2.2–20.7) 0.001	1.9	5.4 (1.9–15.5) 0.002	1.7	2.5 (1.3–4.9) 0.007	22.4 (6.6–76.6) <0.001	4.7 (2.1–10.7) <0.001	6.7 (3.2–14) <0.001	ND
White blood cell count	1 (1.0–1.0) 0.023	*	--	--	--	1 (1.0–1.0) 0.826	1 (1.0–1.0) 0.007	1 (1.0–1.0) 0.637	1 (1.0–1.0) 0.008	1 (1.0–1.0) 0.015
Neutrophil-to-lymphocyte ratio	1.1 (1.1–1.2) 0.001	*	--	--	--	1 (1–1.1) 0.756	1.2 (1.1–1.3) 0.001	1 (0.9–1.1) 0.879	1.1 (1–1.1) 0.004	1.1 (1.1–1.2) <0.001

*eliminated from the model because of in-significance

b: not done.

The AUROC curve of the score for surgery was 0.88 (95% CI of 0.83–0.94, *p*-value <0.001). At a ≥3-point cutoff value, the model had 71.8% sensitivity and 88.5% specificity, whereas the positive likelihood ratio was 6.22 (95% CI, 3.31–11.68) and the negative likelihood ratio was 0.32 (95% CI, 0.22–0.46) in the prediction of surgery. The goodness-of-fit for the final logistic regression model was assessed using the Hosmer–Lemeshow test. Thus, we found that *p*-value was 0.191 (>0.05), indicating the proper model fitting. Additionally, we observed a positive association between bullae or blister formation, GI effects, or fever and tissue swelling grade and the direction of association was not changed in the final multivariate logistic regression model (Table 5) suggesting that the model assumption was not violated.

All the required laboratory indicators to construct the LRINEC score (i.e., WBC, Hgb, CRP, sodium, creatinine, and glucose; see S2 Table) were available together in 64 patients. The AUROC curve of the LRINEC score for NSTI was 0.55 (95% CI, 0.4–0.69), with an insignificant *p*-value of 0.52.

## Discussion

*N*. *atra* has distinct biological features that makes visual identification relatively easy [6,24]. The clinical manifestations caused by *N*. *atra* envenoming, however, overlap with those of the common crotaline snakebites [11,14,25]. Therefore, in this study we only included patients with a definite diagnosis and those who saw the culprit snake to construct the surgical risk score and excluded those with snakebites for which the culprit snake could not be identified (i.e., clinical diagnosis [4]).

Only a few studies on the risk factors for surgery in snakebites from a single species or genus exist. These factors were finger as the bite site and bullae or blister formation in *P*. *mucrosquamatus* bites analyzed with multivariate logistic regression [14] and finger as the bite site, blister and abscess formation, and venom-induced coagulopathy in *Bothrops* spp. bites analyzed with chi-square analysis [26]. The risk factors for wound necrosis and, therefore, possible surgical intervention were finger or toe as the bite site, bullae or blister formation, and venom-induced coagulopathy in green pit viper (*Trimeresurus* spp.) bites in Thailand [27] and cold packing as the first aid, bullae and blister formation, and suspected wound infection in *T*. *s*. *stejnegeri* bites; both studies were analyzed using multivariate logistic regression [25]. Another study has found risk factors of ecchymosis and cyanosis in rattlesnake bites in North America, analyzed as relative risk assessment [28]. Our study demonstrated that lower limb as the bite site, a ≥3 swelling grade, bullae or blister formation, GI effects, and fever were significantly associated with surgery in *N*. *atra* bites.

From the clinical perspective, these effects appeared quickly after the bite and before surgery. The predictive value for surgery is acceptable, with a ≥3-point risk score. In addition, to avoid determining surgical risk mainly for ACS, which had been reported as extremely high (i.e., 6.6%–39.0%) in Taiwan patients with snakebites [8,29–31], we comprehensively reviewed the medical records of eight patients with suspected ACS, finding that seven had wound necrosis and suspected wound infection before fasciotomy or fasciectomy; hence, we included these in our analysis. We did not incorporate wound necrosis and secondary infection as a predictor in the model, as these already were surgical indications [4,14].

We also found a positive association between bullae or blister formation, GI effects, or fever and swelling grade. Swelling is generally accepted as one measurement of the severity of snakebites [15,32,33]; in *Vipera* spp. bites, the swelling grade is correlated with the serum venom level [34,35]. *N*. *atra* venom consists of a complex mixture of proteins and peptides, mainly cardiotoxins (CTXs, 52.9%–59.4%), neurotoxins (NTXs, 20.5%–23.5%), phospholipase A_2_s (PLA_2_s, 14%–16.8%), cysteine-rich secretory proteins (CRISPs, 2.2%–2.4%), snake venom metalloproteinases (SVMPs, 1.3%–1.7%), and other trace constituents [36,37]. Wound swelling, pain, and necrosis are probably induced by the synergistic actions of CTXs, PLA_2_s [38–42], and other cytotoxic enzymes (e.g., CRISPs) [36,43]; however, the mechanisms of bullae or blister formation are poorly understood, which may be partly induced by the proteolytic effects of SVMPs in the skin [44].

Mao et al. have suggested removing the bullae or blisters to reduce the venom load in the bite wound [14] because venom has been detected in the bullae fluids after the *P*. *mucrosquamatus* bite [45]. Coincidentally, the venom proteins of the *Naja* species (i.e., CTXs and PLA_2_s) have been detected in the bullae fluids after the bite [46]. Even though the current snakebite management guideline recommends that these bullae or blisters should be kept intact and aspirated only if they threaten to rupture [17], the bullae or blisters in *N*. *atra* bites are almost always developed in patients with wound necrosis, so early removal likely will be helpful for better wound inspection and to reduce the toxin load, similar to the management of chemical burns [47].

Notably, a 57-year-old male patient who was bitten by an *N*. *atra* on his left foot had repeated vomiting and diarrhea within 1 h post-bite, developing bullae and blisters, wound necrosis, lymphangitis and lymphadenitis, fever, and necrotizing fasciitis requiring multiple surgeries. His blood and bullae fluid cultures before initial surgery yielded *S*. *algae* and *S*. *algae* and *M*. *morganii*, respectively. Bacteria may have a role in the formation of bullae or blisters [48]. Furthermore, we have provided evidence that *Shewanella* soft tissue infection is a route of bacteremia transmission [49,50].

GI effects were reported only in *N*. *atra* bites, in contrast to the other common crotaline snakebites (i.e., *T*. *s*. *stejnegeri* and *P*. *mucrosquamatus*) in Taiwan [4,14,25]. Typically, patients presented with repeated vomiting and diarrhea in the first few hours post-bite before treatment and promptly responded to the administration of specific antivenom and/or anti-emetics [4]. Muscarinic PLA_2_s and/or CTXs could be involved [51,52].

However, fever, which also correlates with swelling grade, is more likely to be caused by multiple factors, including snakebite-related tissue inflammation and/or infection [3,15]. Pyrogenic response to the administration of antivenom is less favorable, as that has not been reported in the past 20 years in Taiwan [53]. Moreover, this response has not been observed in *B*. *m*. *multicinctus* bites that cause minimal tissue reaction, and patients receive the same bivalent specific antivenom for *N*. *atra* and *B*. *m*. *multicinctus* bites [54]. Therefore, we believe that fever is an important feature of *N*. *atra* bites and suggest that the presence of GI effects and fever are caused by high venom inoculation and, hence, more tissue swelling and/or wound complications (i.e., wound necrosis, NSTI, and surgery).

The reasons for increased risk for surgery in lower limbs as the bite sites for *N*. *atra* bite remain unknown. We suspect a higher likelihood of wound contamination (e.g., water and soils) and a secondary infection in lower limb than in other anatomic regions.

We observed a high incidence of NSTI following *N*. *atra* bites. The diagnosis of NSTI requires prompt surgical exploration in the cases of high clinical suspicion, especially when a patient’s pain is out of proportion [18] and the patient is unresponsive to the administration of antivenom [55,56]. NSTI’s histopathology findings include necrosis of the superficial fascia; polymorphonuclear WBCs in the deep dermis and fascia; edema of the reticular dermis, subcutaneous fat, and superficial fascia; fascial artery and vein fibrinous involvement; and visible bacteria on the gram stain of the fascia and dermis [57,58].

The patients in our study supported the diagnosis of snakebite-induced NSTI. However, a median time of 8.6 h between NSTI diagnosis and surgery has been reported [59], contradicting our study’s findings. Our patients’ surgeries were delayed, likely because of the aggressiveness of the pathogens and the relatively healthy status of the patients with *N*. *atra* bites [13,60]. NSTI has been described as three microbiological classes based on the intraoperative bacterial culture findings [61]: Type I (polymicrobial), Type II (monomicrobial), and Type III (clostridial myonecrosis) [61,62].

We found that the most common pathogens (i.e., *M*. *morganii* and *Enterococcus* spp.) isolated during the initial surgery differed from those in Type I NSTI [63,64]. These pathogens were also identified in the oropharynx of *N*. *atra* [13,65,66], which suggested that they may come from the snake’s mouth during envenoming. In addition, a few pathogens were identified only in the later surgeries, suggesting secondary colonization or nosocomial infection. This information may have therapeutic implications for clinicians tailoring antibiotic administration at different periods post-bite.

We further examined the association between the laboratory data (i.e., WBC, NLR [22], and LRINEC score [21]) and surgery or NSTI [4]. However, these parameters obtained on arrival in the ED failed to predict surgery after being adjusted with the clinical risk factors, probably because the predictive role of these non-specific inflammatory markers was mediated through these factors. In addition, the progress of bacterial inoculation to infection is dynamic [67]. Serial laboratory data could be better correlated with the infection continuum than only with a single data set obtained on patients’ arrival.

The Taiwan government produces four types of antivenom against six venomous snakebites: a bivalent for *N*. *atra* and *B*. *m*. *multicinctus*, a bivalent for *T*. *s*. *stejnegeri* and *P*. *mucrosquamatus*, a monovalent for *D*. *acutus*, and a monovalent for *D*. *siamensis*. These formulas have not changed since the 1990s, and they all are ammonium sulfate-precipitated F(ab′)_2_ fragments in a lyophilized form [6,68].

According to the position statement from the Taiwan Poison Control Center (PCC), 6–10 vials are recommended to treat an envenomed patient, a figure based on the average amount of venom milked from a cobra (mean, 48 or 217 mg for the western and eastern types of *N*. *atra*, respectively) and the neutralizing activity of the antivenom (8.58 or 17.42 mg per vial, respectively) [6]. Our findings are consistent with those of a previous study and the PCC’s suggestion that the median antivenom dose of 10 vials be administered [4]. However, a recent rodent study has demonstrated that the bivalent antivenom was insufficient to neutralize the cytotoxicity produced by CTXs [69]. Prospective evaluations of the optimal dosages of antivenom to counteract the synergistic cytotoxic effects of the enzymes that participate in the induction of wound necrosis and a better design of antibodies and medications against CTXs in *N*. *atra* or other cytotoxic *Naja* bites are urgently required [70].

### Study limitations

This study has several limitations. First, because *N*. *atra* bites are uncommon in Taiwan, a major limitation is the lack of a second population to validate the surgical risk score. The inclusion of suspected cases also raises the possibility of some of them not having been bitten by cobras since it is widely accepted that the identification of the culprit snake by the patient is usually imprecise. Nonetheless, the comparison between definite and suspected cases (Table 1) did not show significant differences, which is in support of a correct diagnosis.

Second, the wound bacteriology might have been underestimated because we uncovered only 18 bacterial species compared to 23 in Mao et al.’s study [3]. Possible reasons include the lower number of bacterial species in the deep tissue cultures than in the wound swab sampling, the fastidious or uncultivable bacteria, and the several treatment forms (e.g., wound cleansing and topical herb application) initiated before the sampling. In addition, all patients received antibiotics before obtaining the surgical samples, which might have changed the bacterial loads and complexity. Therefore, we suggest a next-generation sequencing technique to better discriminate wound bacteriology [13]. Nevertheless, we reported only deep tissue cultures, which generally are accepted as the gold standard to diagnose wound infection and as guidance for further antibiotics administration [71].

Third, the histopathological examination of the debrided tissue in the *N*. *atra* bites was not routinely performed at our hospital. The debrided tissues did not always contain all layer parts for examination, and the specific changes in tissue layers due to various pathogens could not be demonstrated clearly [58,72]. Moreover, the biological mechanisms of the venom-induced tissue injury remains undetermined [42].

Finally, the data of the study were extracted from medical charts that cover a long period of time, and this introduces the possibility of variations in the way the information has been introduced into the charts; that is, different physicians may introduce a different degree of detail in the charts. Additionally, it is likely that different physicians were involved in the therapeutic and surgical decisions in these patients, with the likelihood of having differences in the trends to do or not to do surgery at different times. Because this study is retrospective, which has certain inherent limitations, the results should be interpreted cautiously. Nevertheless, ours is the first study to investigate the risk factors for surgery for *N*. *atra* bites, which can cause serious wound complications in Taiwan.

## Conclusions

Although *N*. *atra* bites are relatively uncommon in Taiwan, they can cause serious local wound complications in contrast to the common crotaline snakebites (i.e., *T*. *s*. *stejnegeri* and *P*. *mucrosquamatus*), thus, requiring repetitive debridement and reconstructive surgeries to restore limb function. Our study suggested that lower limb as the bite site, ≥3 swelling grade, bullae or blister formation, GI effects, and fever are risk factors for surgery. We further analyzed wound bacteriology during the initial surgery and provided histopathological evidence for NSTI. These findings can be helpful for patient management and disposition following an *N*. *atra* bite. When used alone, common laboratory parameters, although helpful for measuring inflammation and infection, may not accurately predict the outcomes of *N*. *atra* bites.

## Supporting information

S1 Table Univariate logistic regression analysis of risk factors associated with surgery in 161 *Naja atra* envenomed patients. (DOCX)

S2 Table Laboratory findings in NSTI and non-NSTI groups. (DOCX)
