# Correction: The development of surgical risk score and evaluation of necrotizing soft tissue infection in 161 *Naja atra* envenomed patients

**DOI:** 10.1371/journal.pntd.0011035

**Published:** 2023-01-04

**Authors:** Chih-Sheng Lai, Po-Yu Liu, Chi-Hsin Lee, Cheng-Hsuan Ho, Wei-Ling Chen, Kuo-Lung Lai, Hung-Yuan Su, Wen-Loung Lin, Kuo-Chen Chung, Yi-Yuan Yang, Chung-Wei You, Kuang-Ting Chen, Yan-Chiao Mao

There are errors in the author affiliations. The correct affiliations are as follows: Chih-Sheng Lai^1,2^, Po-Yu Liu^3,4^, Chi-Hsin Lee^5,6,7^, Cheng-Hsuan Ho^8,9^, Wei-Ling Chen^9,10,11^, Kuo-Lung Lai^12^, Hung-Yuan Su^13,14^, Wen-Loung Lin^15^, Kuo-Chen Chung^16^, Yi-Yuan Yang^5,6,7^, Chung-Wei You^17^, Kuang‐Ting Chen^18^, Yan-Chiao Mao^2,9,11*^

**1** Division of Plastic and Reconstructive Surgery, Department of Surgery, Taichung Veterans General Hospital, Taichung, Taiwan, **2** College of Medicine, National Chung Hsing University, Taichung, Taiwan, **3** Division of Infectious Diseases, Department of Internal Medicine, Taichung Veterans General Hospital, Taichung, Taiwan, **4** Rong Hsing Research Center for Translational Medicine, National Chung Hsing University, **5** School of Medical Laboratory Science and Biotechnology, College of Medical Science and Technology, Taipei Medical University, Taipei, Taiwan, **6** PhD Program in Medical Biotechnology, College of Medical Science and Technology, Taipei Medical University, Taipei, Taiwan, **7** Core Laboratory of Antibody Generation and Research, Taipei Medical University, Taipei, Taiwan, **8** Department of Emergency Medicine, Tri-Service General Hospital, Taipei, Taiwan, **9** School of Medicine, National Defense Medical Center, Taipei, Taiwan, **10** Psychiatry Department, Chiayi Branch, Taichung Veterans General Hospital, Chiayi, Taiwan, **11** Division of Clinical Toxicology, Department of Emergency Medicine, Taichung Veterans General Hospital, Taichung, Taiwan, **12** Division of Allergy, Immunology and Rheumatology, Department of Internal Medicine, Taichung Veterans General Hospital, Taichung, Taiwan, **13** Department of Emergency Medicine, E-Da Hospital and I-Shou University, Kaohsiung, Taiwan, **14** The School of Chinese Medicine for Post Baccalaureate, I-Shou University, Kaohsiung, Taiwan, **15** Taichung Wildlife Conservation Group, Taichung, Taiwan, **16** Division of Traumatology, Department of Emergency Medicine, Taichung Veterans General Hospital, Taichung, Taiwan, **17** Green Nature Experience, New Taipei, Taiwan, **18** Show Chwan Memorial Hospital, Changhua, Taiwan
