# Supplementary material for: The development of surgical risk score and evaluation of necrotizing soft tissue infection in 161 Naja atra envenomed patients
Source: PLoS Negl Trop Dis. 2022 Feb 10;16(2):e0010066. doi: 10.1371/journal.pntd.0010066 (PMC8830662; doi:10.1371/journal.pntd.0010066)
Supplement: S1 Table — (DOCX) [file pntd.0010066.s001.docx]

| **S1 Table. Univariate logistic regression analysis of risk factors associated with surgery in 161 *Naja atra* envenomed patients** | | |
| --- | --- | --- |
|  | Crude OR (95% CI) | *p*-value |
| Male | 0.7 (0.4-1.4) | 0.378 |
| Age (yr) | 1.0 (1.0-1.0) | 0.235 |
| Body part bitten |  |  |
| Upper limb | 0.4 (0.2-0.7) | 0.001 |
| Lower limb | 3.0 (1.6-5.7) | 0.001 |
| Others (neck or trunk) |  |  |
| First aid |  |  |
| Rope binding^a^ | 2.2 (0.8-6.2) | 0.134 |
| Incision and suction | 0.3 (0.1-1.6) | 0.172 |
| Topical herbs | 1.4 (0.3-6.3) | 0.688 |
| Cold packs | 1.8 (0.5-6.6) | 0.344 |
| Alcohol ingestion | 1.0 (0.2-5.2) | 0.988 |
| Comorbidity |  |  |
| Diabetes mellitus | 0.9 (0.3-2.3) | 0.829 |
| Liver disease | 1.2 (0.4-3.4) | 0.767 |
| Vascular disease (i.e., CAD, CVA) | 1.4 (0.3-6.3) | 0.688 |
| Malignancies | 2.1 (0.2-23.1) | 0.561 |
| Clinical manifestations |  |  |
| Tissue swelling grade (1-4)^b^ | 4.4 (2.7-7.2) | <0.001 |
| <3 | Reference |  |
| ≥3 | 11 (5-24.3) | <0.001 |
| Acute Compartment syndrome, suspected | 7.7 (0.9-63.9) | 0.06 |
| Wound necrosis^c^ | -- | -- |
| Bullae or blister formation | 10.8 (3.6-32.6) | <0.001 |
| Local numbness | 1 (0.5-2.1) | 1 |
| Lymphangitis or lymphadenitis | 1.8 (0.5-6.5) | 0.354 |
| Necrotizing soft tissue infection^d^ | -- | -- |
| Necrotizing fasciitis |  |  |
| Necrotizing adipositis |  |  |
| Finger or toe wet gangrene |  |  |
| Fever (≥38℃) | 16.1 (6.6-39.6) | <0.001 |
| Gastrointestinal effects (i.e., vomiting or diarrhea) | 6.7 (3.1-14.5) | <0.001 |
| Ptosis or muscle weakness | 7.7 (0.9-63.9) | 0.06 |
| Laboratory findings |  |  |
| Blood tests |  |  |
| White blood cell counts, x 10^9^/L | 1 (1.0-1.0) | 0.023 |
| Neutrophil to lymphocyte ratio | 1.1 (1.1-1.2) | 0.001 |
| Hemoglobin, g/dL | 1.0 (0.9-1.2) | 0.859 |
| C-reactive protein, mg/dL | 1.4 (1-1.9) | 0.057 |
| Serum sodium, mEq/L | 1.1 (1-1.2) | 0.207 |
| Serum creatinine, mg/dL | 1.5 (0.5-4.5) | 0.442 |
| Blood glucose, mg/dL | 1 (1-1.0) | 0.8 |
| Blood creatine kinase (CK), U/L | 1.0 (1.0-1.0) | 0.086 |
| a: included any form of rope, rubber band, or towel/clothes bindings; b: 3 bitten site other than limbs and 1 swelling grade missed were excluded; c: not analyzed because it is the surgical indication; d: it is the surgical indication and diagnosis. | | |
