# Supplementary material for: The development of surgical risk score and evaluation of necrotizing soft tissue infection in 161 Naja atra envenomed patients
Source: PLoS Negl Trop Dis. 2022 Feb 10;16(2):e0010066. doi: 10.1371/journal.pntd.0010066 (PMC8830662; doi:10.1371/journal.pntd.0010066)
Supplement: S2 Table — (DOCX) [file pntd.0010066.s002.docx]

| **S2 Table. Laboratory findings in non-NSTI and NSTI groups** | | | | |
| --- | --- | --- | --- | --- |
|  | Non-NSTI (n = 89) | NSTI (n = 72) | Total cases ( n = 161) | *p*-value |
| White blood cell counts, x 10^9^/L | 7800 (6300-10500) | 9200 (7100-11500) | 8000 (6500-10785) | 0.028 |
| <15 (0 point)^a^ | 81 | 51 | 132 | 0.017 |
| 15-25 (1 point) | 2 | 8 | 10 | 0.017 |
| >25 (2 points) | 0 | 0 | 0 |  |
| Hemoglobin, g/dL | 14 (12.3-15.1) | 13.7 (12.6-15.1) | 13.7 (12.5-15.1) | 0.911 |
| >13.5 (0 point) | 46 | 33 | 79 | 0.952 |
| 11-13.5 (1 point) | 29 | 23 | 52 | 0.622 |
| <11 (2 points) | 8 | 3 | 11 | 0.362 |
| C-reactive protein, mg/dL | 0.2 (0.06-0.8) | 0.8 (0.1-5.79) | 0.28 (0.09-1) | 0.034 |
| <15 (0 point) | 48 | 31 | 79 | 0.028 |
| ≥15 (4 points) | 0 | 4 | 4 | 0.028 |
| Serum sodium, mEq/L | 140 (139-142) | 141 (138-143) | 140 (139-143) | 0.211 |
| ≥135 (0 points) | 78 | 56 | 134 | 1 |
| <135 (2 point) | 3 | 2 | 5 | 1 |
| Serum creatinine, mg/dL | 1 (0.8-1.1) | 0.9 (0.7-1.1) | 1 (0.8-1.1) | 0.84 |
| ≤1.6 (0 points) | 82 | 56 | 138 | 0.307 |
| >1.6 (2 points) | 1 | 3 | 4 | 0.307 |
| Blood glucose, mg/dL | 118 (103-142) | 123 (106-139) | 119 (103-141) | 0.555 |
| ≤180 (0 point) | 51 | 38 | 89 | 0.131 |
| >180 (1 point) | 11 | 3 | 14 | 0.131 |
| LRINEC^b^ score | 1 (0-1) | 1 (0-2) | 1 (0-1) | 0.739 |
| a: point score of LRINEC; b: Laboratory Indicator for Necrotizing Fasciitis | | | | |
